# Phenomenon of Hematocephalus: A Comprehensive Review of the Literature

**DOI:** 10.31662/jmaj.2022-0202

**Published:** 2023-03-13

**Authors:** Martin Susanto, Andre Marolop Pangihutan Siahaan, Beny Atmadja Wirjomartani, Winda Pardede, Ika Riantri

**Affiliations:** 1 University of Sumatera Utara, Medan, Indonesia; 2 University of Padjadjaran, Bandung, Indonesia; 3 Medistra Health Institute, Lubuk Pakam, Indonesia

**Keywords:** hematocephalus, hematocephaly, hemocephalus, management

## Abstract

The phenomenon of hematocephalus is still not fully understood. Intraventricular hemorrhage volume and intracranial pressure play a substantial role in the outcome and survival of the patients. The intraventricular hemorrhage resulting in an increased intracranial pressure is known by the term “hematocephalus.” The mortality rate ranges from 60% to 91% when hemorrhage affects all four ventricles. Even for partial hematocephalus, the mortality rate has been reported to be 32% to 44%. Therefore, the main objective in managing hematocephalus is to remove intraventricular blood efficiently and quickly because doing so will reduce ventricular dilatation and will rebalance cerebrospinal fluid circulation. However, the current standard management, which is inserting a ventricular drain immediately after an intraventricular hemorrhage, appeared to be of little value as the catheters are invariably clogged with blood clots. Long-term outcomes from the external ventricular drainage insertion plus subsequent intraventricular fibrinolytic therapy have been encouraging, but it also carries a substantial risk of new intracranial bleeding. The neuroendoscopic approach was created to aid in the treatment of hematocephalus and to enable the hematoma to be reduced or removed quickly without invasive surgery or the administration of fibrinolytic medications, preventing the intraventricular inflammatory reactions that result from hematoma degradation products. A controlled trial is necessary to ascertain whether this procedure enhances patient outcomes when compared to ventricular draining with or without thrombolysis.

## Introduction

According to Benes, hematocephalus is an intraventricular hemorrhage (IVH) resulting in an increased intracranial pressure (ICP) ^(1)^. The phenomenon of hematocephalus is still not fully understood. Initially, it was considered only the elevation of ICP in the primary or purely intraventricular bleeding. However, later studies concluded that the true offending lesion was intracerebral bleeding to the deep brain structures, along with the shifting of these structures and brainstem compression ^(2)^. The accumulation of blood in the ventricles of the brain plus increased ICP is known as either *hematocephalus* ^(3), (4)^, *hematocephaly* ^(5)^, or *hemocephalus* ^(6)^. This terminology should not be confused with “hydrocephalus” as this condition caused by the buildup of cerebrospinal fluid (CSF) is not appropriate to be applied to patients who have IVH. However, hydrocephalus may develop in the long term after the ventricular blood has been evacuated because of either acute obstruction of the CSF pathways or chronic fibrosis of the leptomeninges ^(6)^.

To date, research regarding the phenomenon of hematocephalus is still limited. This comprehensive literature review aimed to provide information and collect research regarding the presentation, diagnosis, classification, mechanism, current neurosurgical treatment, and prognosis of hematocephalus.

## Methods

We conducted extensive research in the database of PubMed, Google Scholar, EBSCO, DOAJ, and Cochrane. We utilized the terms “hematocephalus” OR “hematocephaly” OR “hemocephalus” to carry out a more thorough study. There were 274 articles found relevant. We took into account research in humans. As a result, we left out 210 studies. Specifically based on presentation, diagnosis, classification, mechanism, current neurosurgical treatment, and prognosis based on the research included in this review, we intended to make a thorough assessment of hematocephalus. Seven articles were excluded because of similarity to other articles, unclear statements, irrelevant correlation with the main topic, or concerning more on other diseases rather than hematocephalus. To add depth to the discussion material, we also added seven books of literature from medical dictionaries and textbooks. In the end, 64 studies were included in this comprehensive review.

## Presentation, Diagnosis, and Classification

The presence of IVH, which was once thought to be always deadly, has long been recognized by clinicians. This assumption was supported by the findings of postmortem studies, which revealed diffuse blood within the ventricular system in individuals with an apoplectic beginning of neurologic deterioration, advancing to coma, brainstem dysfunction, and death. Diagnosis may be performed on live patients with the advancement of computed tomography (CT) scanning, ultrasound sonography (USG), and magnetic resonance imaging (MRI) ^(7), (8), (9), (10)^.

The clinical discovery that primary intracerebral bleeding and cerebral aneurysm rupture frequently followed acute ventricular distension and substantially elevated ICP within a few hours of the ictus was made possible by breakthroughs in neuroradiology and evolving knowledge. The phenomenon substantially complicates the clinical course and is referred to as “acute hydrocephalus” whether or not blood is present in the ventricles. Numerous patients suffer from substantial ventricular bleeding, blood clot blockage, and ventricular tamponade. At autopsy, early acute ventricular distension would have been missed. Continuous CSF production can worsen transtentorial and transforaminal herniation as well as ventricular distension. In the past, many of these patients passed away too soon, with lumbar puncture and secondary pontine hemorrhage occasionally hastening their demise. The understanding of these processes and the neurosurgical management of acute ventricular dilation were vastly enhanced by more evidence. Although some people exhibit true acute hydrocephalus, the term acute hydrocephalus does not apply to those who frequently have IVH ^(6), (11), (12), (13), (14), (15), (16), (17)^.

**Figure 1.**
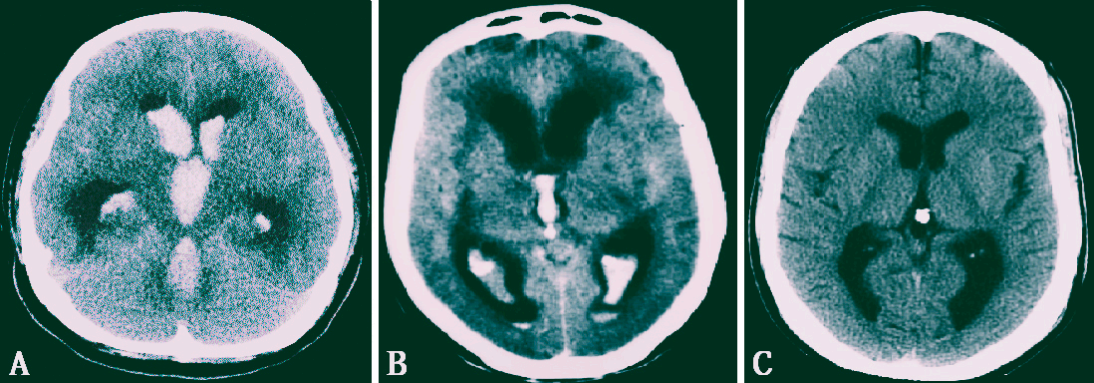
(A) Hematocephalus totalis. (B) Hematocephalus partialis. (C) IVH without a real hematoma.

It is crucial to adhere to Pia’s proposed classification of IVH for diagnostic, therapeutic, and prognostic measures of hematocephalus (Figure 1) ^(18)^:

1) Hematocephalus totalis, which is hematoma throughout the entire ventricular system,

2) Hematocephalus partialis, which occurs when a blood clot only fills specific areas of the ventricular system,

3) IVH without real hematoma.

Using a 12-point grading scale, Graeb et al., who examined IVH, discovered a link between a high IVH grade and a negative outcome. The Graeb grading of IVH, which varies from 4 to 12, is the result of adding the component scores assigned to the lateral ventricles, third ventricle, and fourth ventricle ^(19)^:

a) Lateral ventricles (each lateral ventricle is scored separately).

Score 1 = trace of blood or mild bleeding

　　　2 = less than half of the ventricle filled with blood

　　　3 = more than half of the ventricle filled with blood

　　　4 = ventricle filled with blood and expanded.

b) Third and fourth ventricles (each one is scored separately).

Score 1 = blood present, ventricle size normal

　　　2 = ventricle filled with blood and expanded.

## Mechanism

Hematocephalus results from bleeding into the cerebral ventricles, either from a main intraventricular source or, more commonly, from an intraparenchymal hemorrhage that is close to the ventricular wall, as in situations of hypertonic hemorrhage or aneurysm rupture ^(2), (20)^. Elevated ICP with decreased cerebral perfusion, mechanical disruption, ventricular wall distension, and perhaps an inflammatory response are the plausible processes by which IVH volume affects the outcome ^(21), (22), (23), (24), (25)^.

Hematocephalus used to be thought of as a clinical indication that was harmful or even foreboding. It quickly became clear, nevertheless that primary IVH did not pose a major risk to the individual because blood could be evacuated with a relatively easy puncture. Additionally, advancements in imaging technology made it possible to safely remove intracerebral blood clots in cases of subsequent intraventricular bleeding ^(2)^.

In cases of secondary hematocephalus, the impact of bleeding and clinical status during the acute stage of the disease depended on the amount of blood in the cerebral ventricles as well as the degree of cerebral injury brought on by the bleeding. Massive intraventricular bleeding causes the ventricular space to quickly fill with blood clots, making it impossible for the CSF hemolytic characteristics or the compliance of this fluid-filled region to function. Therefore, hydrocephalus and a subsequent progressive increase in ICP are caused by an abrupt stoppage of CSF circulation ^(2), (26)^.

Additionally, it has been proposed that the direct mass effect of IVH may result in decreased cerebral blood flow ^(10), (27)^. According to studies, the ependymal and subependymal layers of the brain and the brain stem sustain substantial damage as a result of the blood clot itself ^(28)^. According to animal studies, the damage to the surrounding tissues happens independently of the mass effect. The blood, most likely as a result of an inflammatory response, was discovered as the source of subarachnoid fibrosis, substantial ependymal cell loss, and subependymal glial proliferation on the walls of the lateral ventricles ^(29)^.

Previous studies have shown that IVH volume and ICP play a substantial role in outcome and survival ^(30), (31)^. Young et al. investigated the relationship between IVH volume and outcome, revealing that total ventricular volume (TVV) was associated with a poorer outcome and that there was a “lethal volume” of 20 mL ^(30)^. According to a different study, IVH volume predicts mortality irrespective of the Glasgow Coma Scale (GCS) ^(21)^. However, the challenge of measuring the volume of blood in standard clinical practice has hindered the study of IVH volume and its therapeutic application. IVH is more diffuse and encompasses various structures, in contrast to ICH, where the hematoma is more clearly defined and can be approximated using techniques like ABC/2 ^(22), (32)^.

Ziai et al. discovered that after controlling for IVH volumes, age, presentation GCS, and pulse pressure, the proportion of high ICP (>30 mm Hg) recordings per individual was an independent predictor of 30-day death ^(31)^. The relevance of ICP elevation (≥20 mm Hg) in the first 48 h following head injury as a poor prognostic predictor, particularly if ICP is recalcitrant to treatment, is consistent with the research on traumatic brain injury ^(31), (33), (34)^.

Because the compensatory mechanism is weakened, blood clots in the ventricular system cause abrupt CSF blockage and a marked rise in ICP. As the ascending reticular activating fiber system (ARAS) function is impaired by the presence of clots deep within the cortical or ventricular structures, an individual becomes ventilator dependent and needs a lengthy stay in the intensive care unit (ICU). This results in a decreased level of consciousness. The fibrin degradation products (FDPs) and bilirubin oxidation products (BOx) that are created during the breakdown process will enter and circulate in the CSF as the clot within the ventricular system undergoes lysis. Arachnoid granulation will then be reached by FDPs and BOx, which will result in delayed communicating hydrocephalus ^(35)^.

The juvenile thin-walled blood vessels that burst cause bleeding into the subependymal germinal matrix and possibly the lateral ventricles, which causes a condition known as germinal matrix hemorrhage (GMH). In a newborn rat GMH model, recent research showed the disruption of the exchange between CSF and interstitial fluid (ISF) and the development of hydrocephalus on day 28. These effects were partially restored by astrogliosis inhibition. GMH caused astrogliotic scarring, decreased CSF reabsorption through the meningeal lymphatic channels, and inhibited the exchange of CSF and ISF. Scar tissue development hinders the CSF clearance pathway. Glymphatic system dysfunction might be linked with aquaporin-4 redistribution. These waste products build up, as well as more ISF in the brain, as a result of glymphatic system disruption. The brain injury that results from the rupture of a significant blood artery is exacerbated by these occurrences, which are also present before and after subarachnoid bleeding ^(36), (37), (38)^.

## Management

The main objective in managing hematocephalus is to remove intraventricular blood efficiently and quickly because doing so will reduce ventricular dilatation and will rebalance the CSF circulation ^(39)^.

The current standard management, which is inserting a ventricular drain immediately after an IVH, appeared to be of little value as the catheters are invariably clogged with blood clots. However, only the sequelae of hydrocephalus and elevated ICP are addressed by this treatment ^(35), (40), (41), (42)^. Modern microsurgical, stereotactic, and drainage procedures cannot stop the resorption of blood degradation products from creating membranous structures or stenotic lesions in the cerebral ventricles. These mechanisms explain the presence of cysts and posthemorrhagic hydrocephalus ^(2)^.

### External ventricular drainage plus intraventricular fibrinolysis

Another less invasive treatment choice for individuals suffering from severe intraventricular bleeding is the placement of an external ventricular drainage system, followed by intraventricular fibrinolytic therapy with tissue plasminogen activator or urokinase. This approach showed a mortality rate of 6% and a poor-outcome rate of 30%. In cases of secondary IVH brought on by subarachnoid or intracerebral hemorrhage, this method has a success rate of more than 76%. However, when treating an IVH brought on by vascular malformations, this strategy could be risky. Hence, it is typically started only after stabilizing the abnormality with either an endoscopic procedure or an open surgery ^(10), (43), (44), (45), (46), (47), (48)^.

The fibrinolytic system is an enzyme mechanism that breaks down blood clots physiologically. In this system, plasminogen, an inactive proenzyme, can be transformed into plasmin, an active enzyme that breaks down fibrin into soluble FDPs. Tissue plasminogen activator, often known as t-PA, is the most substantial intrinsic plasminogen activator and is produced and secreted by endothelial cells ^(48)^. The unseen fibrinolytic property of CSF is responsible for the persistence of intracranial extravascular blood clots, particularly those in the ventricular system and subarachnoid region. Because t-PA is absent from normal CSF, there is no direct fibrinolytic activity. t-PA is released into the CSF as a result of meningeal irritation, such as bleeding, but the amounts are insufficient to effectively or quickly dissolve clots ^(49)^.

The quantity of blood in the ventricular system is visually estimated to determine the appropriate t-PA dose. The dosage ranges from 3 to 5 mg most frequently, with greater doses (8 mg) administered to comatose individuals whose IVH affects all four ventricles. When practical, a volume of CSF equal to the whole volume of t-PA being injected is withdrawn prior to the intraventricular injection of the drug. A 1 to 1.5 mL flush of preservative-free normal saline is administered after the injection. Then, the EVD is clamped for 2 h while the ICP is closely watched. The EVD is alternately opened and clamped for 1 h at a time for a total of 12 h after the initial 2 h ^(10)^.

Long-term outcomes from the intraventricular injection of fibrinolytic medicines have been encouraging, but it also carries a substantial risk of new intracranial bleeding. This strategy, meanwhile, is potentially risky when treating IVH brought on by vascular malformations, so it is typically employed only after stabilizing the abnormality with endoscopic or open surgery ^(39), (50), (51)^.

### Neuroendoscopic approach

Different types of intracranial bleeding have been treated in the past using rigid or flexible endoscopes, but total blood clearing from all of the ventricles has never been achieved. Surprisingly, the most challenging and time-consuming element of the entire treatment is the aspiration of the clots in the lateral ventricles. The main issue is the necessity for orientation in an area that has been warped by the distension brought on by the hemorrhage without the use of visual cues. The most important orienting structure toward the third ventricle, the choroid plexus, must be visually located once a substantial amount of blood has been removed. The only tool that can navigate to the fourth ventricle is a flexible endoscope. When evaluating the advantages and disadvantages of rigid versus flexible devices, this is undoubtedly crucial. The aqueductal diameter is not an issue because it dilates naturally in all pathological circumstances and can be softly passed through without harming the nervous system. Because vision is relatively dim and hazy and movements inside the ventricles may result in significant cortical and subcortical damage, using rigid endoscopes to treat IVH is particularly damaging. The same logic applies to the microsurgical method, which is not recommended for the treatment of tetraventricular hemorrhage because of its intrusiveness and inability to reach all cavities, although it has potential for success in treating intraparenchymal hematomas ^(39), (52), (53), (54), (55), (56)^.

**Figure 2.**
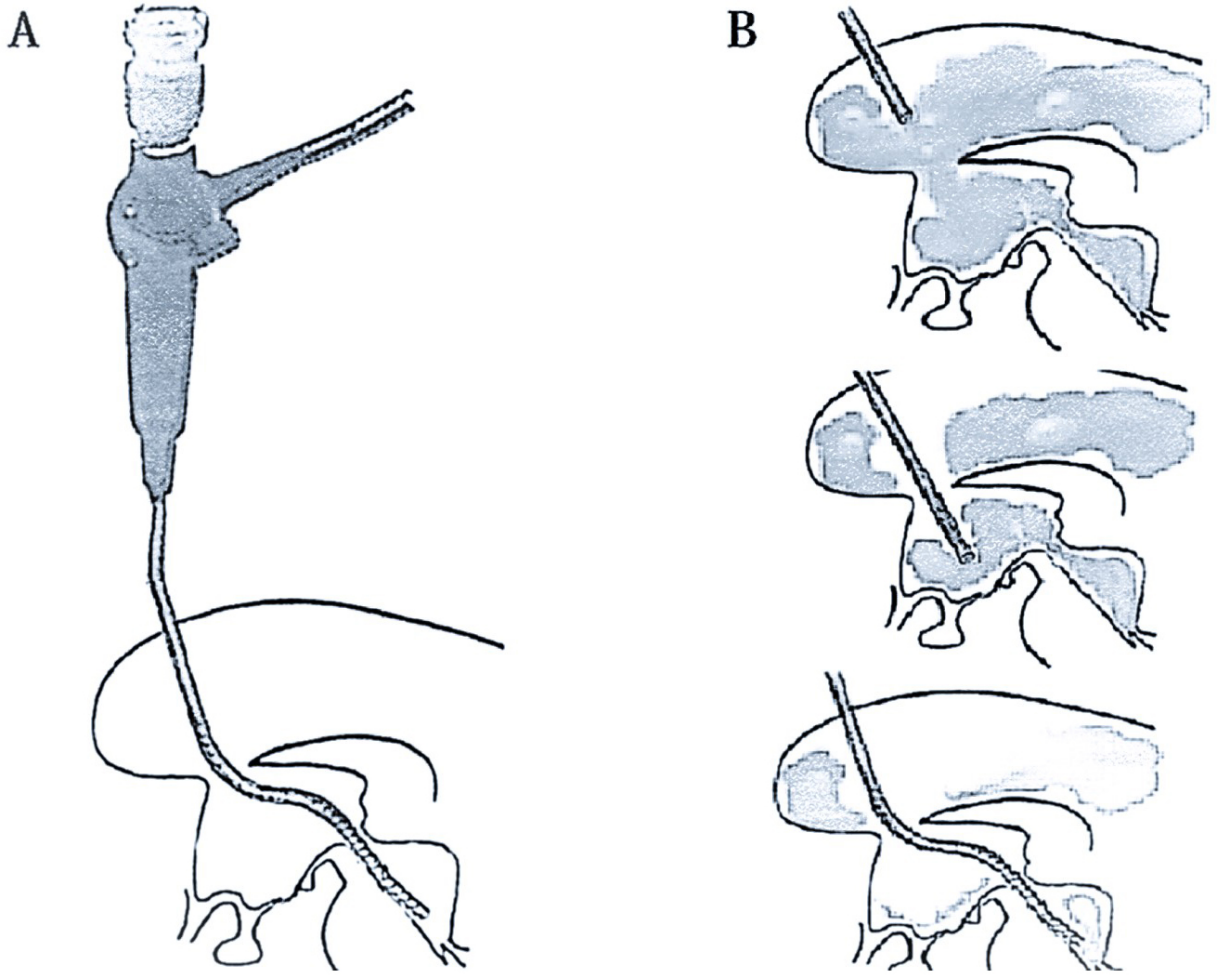
(A) Diagram showing the method of navigating from the lateral ventricle to the fourth ventricle system. (B) The frontal horn, third ventricle, aqueduct, and fourth ventricle are the three phases of neuroendoscopic IVH navigation.

The technique of a neuroendoscopic approach has been suggested, nonetheless, in the idea that early evacuation of an intraventricular and intracerebral hematoma is beneficial ^(57), (58)^. The neuroendoscopic approach was created to aid in the treatment of hematocephalus and to enable the hematoma to be reduced or removed quickly without invasive surgery or the administration of fibrinolytic medications, preventing the intraventricular inflammatory reactions that result from hematoma degradation products ^(51)^. A time-limited procedure known as neuroendoscopic lavage allows the direct imaging of the ventricular system and any bleeding or blood clots while being carried out in sterile settings ^(59), (60)^. The ventricles remain dilated during the process because the intraventricular fluid volume is consistently maintained in balance. With this method, the hematoma itself can be decreased or removed through direct mobilization and aspiration, as well as liquefied blood, hematoma particles, and fibrin fragments. With the exception of the temporal horns, all ventricular compartments can be visualized and washed, and if necessary, draining catheters can be inserted using the neuroendoscope (Figure 2). Owing to a variety of factors, neuroendoscopic ventricular lavage may not always be sufficient. These include membrane and multiloculated hydrocephalus development, hemorrhage-induced impaired CSF resorption, concurrent or acquired aqueductal stenosis or blockage, partial hematoma clearance (residual in the temporal horns), and more. A controlled trial is necessary to ascertain whether this procedure enhances patient outcomes when compared to ventricular draining with or without thrombolysis ^(51)^.

The application of neuroendoscopy in patients with massive intraventricular bleeding has substantially reduced drainage dependency and has produced positive clinical outcomes according to the research by Johnson et al. on the prevalence of shunt dependency and clinical outcomes in individuals with massive intraventricular bleeding treated with endoscopic washout and EVD. The early removal of hematomas, the development of new CSF diversion pathways, and the dilution of the FDPs and BOx that can impair the arachnoid granulations are possible causes of this. The modified Rankin scale score showed a substantial advantage in this investigation, suggesting that neuroendoscopy may produce a functional outcome that is potentially better ^(35)^.

Longatti et al. described treating 25 patients with hematocephalus totalis for removing blood clots in the cerebral ventricles using a flexible endoscope. On the day of admission, the endoscopic evacuation of the intraventricular blood was performed on the majority of the patients. Through the working channel of the endoscope, suction and irrigation were used to remove the clot from the lateral, third, and fourth ventricles. To remove the blood from the fourth ventricle, they maneuvered the endoscope through the Sylvius aqueduct. In all patients, they claimed to have been able to get rid of the majority of intraventricular blood. Postoperatively, ventricular drainage was performed for a median of 8 days. In 12% of patients, a ventriculoperitoneal shunt was required. There was no mortality associated with surgery. After the operation, the average stay in the ICU was 18 ± 12 days. The long-term mortality rate (>6 months) was 24%, whereas the short-term mortality rate (1 month) was 12%. 40% of patients resulted in full recovery (GCS score of 5) ^(39)^.

Novak et al. reported the outcomes of endoscopic treatment for primary and secondary hematocephalus in a group of 23 patients using the Wolf endoscopic system in conjunction with frame-based navigation in a virtual reality environment. The endoscopic third ventriculostomy was performed to reduce the amount of blood in the basal cisterns and to restore the patency of the CSF pathways, allowing lumbar puncture to be performed safely to facilitate the process of removing blood from the CSF in the majority of the treated patients. Septostomy was added to allow the revision of both lateral ventricles to be performed using a single approach. The clinical situation enhanced without the necessity for the placement of long-term drainage systems. One patient’s fatal outcome and the negative clinical outcomes in the other three individuals were unrelated to the endoscopic procedure ^(2)^.

According to a case study by Spazzapan et al., a neuroendoscopic ventricular lavage proved effective in treating a neonate with hematocephalus. The patient was born at 32 weeks gestation, weighed 1,740 g, had a head circumference of 31 cm, and achieved 9/10 on the Apgar score. An EVD was initially inserted 14 days after birth to relieve the hematocephalus and stop additional head enlargement due to the increasing head enlargement. After receiving treatment with the EVD for 14 days, the clot still existed and the neonate underwent a complete neuroendoscopic clot removal and lavage because the CSF was hemorrhagic. A ventriculosubgaleal shunt was left behind as a temporary drainage device after the endoscopic treatment went well. Within a week of surgery, the ventricular system began to gradually but steadily enlarge, necessitating repeated punctures of the subgaleal pocket before the CSF was sufficiently clear to allow the placement of a permanent ventriculoperitoneal drain (VPD). No signs of CSF infection were seen. The neonate was discharged after this treatment ^(51)^.

## Prognosis

When hemorrhage extends into all four ventricles, the mortality rate has been 60% to 91%. Even for partial hematocephalus, the mortality rate has been reported to be 32% to 44%. According to Hallevi et al., there is a TVV cut-off of 40 mL above which individuals are 41 times more likely to have a poor outcome and a 50 mL “poor-outcome threshold” above which 100% of individuals have a poor outcome. Similar to other thresholds, TVV > 60 mL was established as the cut-off for mortality. However, the “lethal volume” at which TVV >60 mL had a 60% mortality rate was not identified. In general, the underlying disease, the location of the cerebral hematoma (in secondary IVH), and the severity of the intraventricular bleeding all have a substantial role in the morbidity and mortality of IVH individuals. Primary IVH survivors do frequently experience substantial morbidity. Compared to individuals with primary IVH, individuals with secondary IVH had worse outcomes. Individuals who have hematomas in the thalamus suffer the worst in terms of the location of the hematoma that is producing the IVH. The degree of ventricular dilatation, initial GCS, and elevated ICP are additional indicators of a poor outcome. With conservative therapy, the mortality rate for substantial intraventricular bleeding (Graeb >6) is close to 50%-75% ^(8), (9), (10), (19), (22), (30), (42), (44), (61), (62), (63), (64)^.

## Conclusion

The phenomenon of hematocephalus is still not fully understood. The mortality rate ranges from 60% to 91% when hemorrhage affects all four ventricles. Even for partial hematocephalus, the mortality rate has been reported to be 32% to 44%. However, the current standard management, which is inserting a ventricular drain immediately after an IVH, appeared to be of little value as the catheters are invariably clogged with blood clots. Long-term outcomes from the EVD insertion plus subsequent intraventricular fibrinolytic therapy have been encouraging, but it also carries a substantial risk of new intracranial bleeding. The neuroendoscopic approach was created to aid in the treatment of hematocephalus and to enable the hematoma to be reduced or removed quickly without invasive surgery or the administration of fibrinolytic medications, preventing the intraventricular inflammatory reactions that result from hematoma degradation products. A controlled trial is necessary to ascertain whether this procedure enhances patient outcomes when compared to ventricular draining with or without thrombolysis.

## Article Information

### Conflicts of Interest

None

### Acknowledgement

The authors would like to thank PubMed, Google Scholar, EBSCO, DOAJ, and Cochrane for the accessibility and wide publication of the journals, which are collected and analyzed in this study.

### Author Contributions

All authors contributed to the study conception and design, performed data collection, made substantial contributions to the analyses and interpretation of the data, and wrote this manuscript. All authors read and approved the final manuscript.

### Ethical Approval and Consent to Participate

Not applicable.

### Consent for Publication

Not applicable.

### Availability of Data and Materials

Not applicable.
